# Flat plate solar collector performance using alumina nanofluids: Experimental characterization and efficiency tests

**DOI:** 10.1371/journal.pone.0212260

**Published:** 2019-02-22

**Authors:** Rosa Mondragón, Daniel Sánchez, Ramón Cabello, Rodrigo Llopis, J. Enrique Juliá

**Affiliations:** Departamento de Ingeniería Mecánica y Construcción, Universitat Jaume I, Castellón de la Plana, Spain; Tongji University, CHINA

## Abstract

Solar energy has become an important renewable energy source for reducing the use of fossil fuels and to mitigate global warming, for which solar collectors constitute a technology that is to be promoted. The use of nanofluids can increase the efficiency of solar into thermal energy conversion in solar collectors. Experimental values for the specific heat, thermal conductivity and viscosity of alumina/water nanofluids are needed to evaluate the influence of the solid content (from 0.25 to 5 v%) and the flow rate on the Reynolds, Nusselt and the heat transfer coefficient. In the laminar flow regime, thermal conductivity enhancement over specific heat decrement is key parameter, and a 2.34% increase in the heat transfer coefficient is theoretically obtained for 1 v% alumina nanofluid. To corroborate the results, experimental tests were run in a flat plate solar collector. A reduction in efficiency from 47% to 41.5% and a decrease in the heat removal factor were obtained using the nanofluid due to the formation of a nanoparticle deposition layer adding an addition thermal resistance to heat transfer. Nanofluids are recommended only if the nanoparticle concentration is high enough to enhance thermal conductivity, but no so high so as to avoid wall deposition.

## Nomenclature

**Table:** 

*A*_*C*_	surface area of the solar collector	(m^2^)
*c*_*p*_	specific heat	(J·kg^-1^·K^-1^)
*D*	diameter	(m)
*F*_*R*_	heat removal factor	(-)
*G*_*T*_	global solar radiation	(W·m^-2^)
*h*	heat transfer coefficient	(W·m^-2^·K^-1^)
*k*	thermal conductivity	(W·m^-1^·K^-1^)
*L*	tube length	(m)
$\dot{m}$	mass flow rate of the fluid	(kg·s^-1^)
*Q*_*u*_	rate of useful energy gained	(W)
*T*	temperature	(°C)
*U*_*L*_	overall loss efficiency	(-)
*v*	velocity	(m·s^-1^)
*ρ*	density	(kg·m^-3^)
*ϕ*	solid volume fraction	(-)
*ϕ*_*m*_	maximum packing fraction	(-)
*η*_*i*_	instantaneous collector efficiency	(-)
*μ*	viscosity	(Pa·s)
(*τα*)	absorptance-transmittance product	(-)
*Nu*	Nusselt number	(-)
*Pr*	Prandtl number	(-)
*Re*	Reynolds number	(-)

## Subscripts

**Table:** 

*a*	ambient
*bf*	base fluid
*i*	inlet
*nf*	nanofluid
*o*	outet
*p*	nanoparticle

## Introduction

Increase in the energy global demands and the use of non-renewable energy sources like fossil fuels have reduced the availability of these sources and have produced strong negative environmental effects, such as air pollution and global warming. In order to mitigate these inconveniences, research works have focused on improving the efficiency of technologies using renewable energy sources like solar energy [1,2]. Solar energy is one of the cleanest and cheapest energy resources that can be converted into thermal and electrical energy that is ecofriendly.

Solar collectors are used to convert solar energy into thermal energy using a heat exchanging fluid. The collector absorbs solar radiation by an absorber plate and transfers heat to the absorber fluid by, thus, increasing its internal energy, which can be used for further applications. Among solar collectors, flat plate solar collectors (FPSC) are used within the 40–100°C range, with no optical concentration. Their simplicity, easy maintenance and low operating costs make them suitable for domestic applications. The working fluids used as absorbers are mainly water and mixtures of water and ethylene glycol, but the main drawback of these conventional fluids is their poor thermal properties as they confer the conversion process poor thermal efficiency.

One of the actions that has attracted attention in the last few years to improve the thermal efficiency of this technology is to change conventional working fluids to nanofluids. Nanofluids are stable suspensions of solid particles whose sizes are below 100 nm [3]. These suspensions present larger specific surfaces than conventional colloidal suspensions and are more stable than conventional slurries. Addition of solid particles with thermal conductivity above that of the base fluid has been demonstrated to provide thermal conductivity enhancement and to, thus, increase both the heat transfer coefficient and nanofluid performance [4–9].

Experimental and theoretical studies on the use of nanofluids in FPSCs were carried out. Nanofluids containing alumina, carbon nanotubes, titania, cerium oxide and tungsten trioxide dispersed in water were prepared by the two-step method [10–18]. In these works, the collector’s thermal efficiency was evaluated following the ASHRAE standard at a constant flow rate for different solid concentrations. Enhancement was achieved when nanofluids were used under some experimental conditions. Also, Computational Fluid Dynamics (CFD) simulations were performed to numerically predict thermal efficiency, which well agreed with the experimental results. However, very dilute nanofluids were used in them all (concentrations below 0.4 wt%) and the properties of the nanofluids (specific heat and thermal conductivity) were calculated by existing models. In any case, the thermo-physical properties of the nanofluids were experimentally measured and evaluated.

Several models have been developed to simulate the efficiency of an FPSC for different nanofluids. Bazdidi-Tehrani *et al*. [19] proposed a three-dimensional model to evaluate the turbulent forced convection of titania/water nanofluids, and efficiency enhancement was proved for more concentrated nanofluids (3.16 v%) at a constant Reynolds number. Genc *et al*. [20] proposed a two-dimensional model by introducing a transient heat transfer approach to demonstrate the effect of the thermo-physical properties of alumina/water nanofluids (1–3 v%) at different Reynolds numbers. According to the obtained results, nanofluids can increase thermal efficiency at lower flow rates below a critical value. Purohit *et al*. [21] simulated the thermal efficiency of an FPSC using alumina/water nanofluids (1–6 v%) in laminar flow at constant pumping power. These authors concluded that efficiency increased at constant Reynolds basis, but decreased at constant pumping power. In all these numerical studies, the thermo-physical properties of the nanofluids were obtained from either the existing models or previous works, and no experimental validation in a solar collector was done.

Finally, the thermal efficiency of a different solar collector type, like the U-Tube solar collector, was experimentally measured using alumina, zinc oxide and carbon nanotubes [22–24]. As in previous experimental studies for FPSCs, thermal efficiency was evaluated following the ASHRAE standard using values calculated for specific heat and thermal conductivity. Tests were carried out at constant flow rate for the different solid concentrations and enhancement was achieved when nanofluids were used under some experimental conditions.

The main drawback as to using nanofluids in solar collectors that came over in the literature review was not experimentally measurement the properties of the actually tested nanofluids in solar facilities. In the reviewed works, specific heat, thermal conductivity and viscosity were obtained mainly from existing models. The evolution of both specific heat and thermal conductivity with the solid content can be predicted with existing models in a wide range of concentrations. However, viscosity can be modeled only by Einstein equation for many diluted nanofluids, while it stops increasing linearly and different models are needed when higher concentrations are employed. Therefore, viscosity is the most important property that influences the Reynolds number and the heat transfer coefficient, and experimental results are required.

In this work the efficiency of a flat plate solar collector using a commercially available alumina/water nanofluid was analyzed and compared to the results obtained for the theoretical evaluation of the heat transfer coefficient, calculated by using the nanofluid thermophysical properties previously measured experimentally. In this way, the main purpose of this work is to provide a route to predict the performance of a flat plate solar collector using nanofluids under different experimental conditions from their measured thermal conductivity, specific heat and viscosity.

In the Results section, first the thermo-physical properties previously measured within a wide range of solid contents and temperatures were modeled so that the specific heat, thermal conductivity and viscosity of the nanofluid were obtained at the working temperature. Then thermal behavior was evaluated at different volume fractions and flow rates through the evolution of Reynolds, Nusselt and the heat transfer coefficient. In the laminar flow regime, the Nusselt number was scarcely affected by the solid content and the heat transfer coefficient only increased by 2.34% for the alumina nanofluid containing 1 v% of nanoparticles. Finally, the results of the experimental tests run with an FPSC using the nanofluid at 1 v% are shown, and they were compared to the theoretical evaluation of the heat transfer coefficient. Thermal efficiency was measured and a reduction took place as a result of the reduction in the overall heat transfer coefficient because of solid layer deposition.

## Materials and characterization

A commercial Aerodisp W925 alumina nanofluid, supplied by Degussa, was used for the experimental tests. This nanofluid contains alumina nanoparticles with a primary particle size of 11 nm dispersed in water and electrostatically stabilized at pH = 4. In order to prepare nanofluids at different volume fractions, the original one was diluted with the required amount of distilled water and the pH value was adjusted with HCl 2.75M.

In a previous work by the authors [4], several commercial and non-commercial nanofluids containing alumina, silica and carbon nanotubes were characterized. Of them, the commercial alumina nanofluid was chosen because it was a commercially available nanofluid that presented the most marked increase in thermal conductivity with a slight increase in viscosity.

In this previous work, the stability and the thermo-physical properties of the nanofluid were experimentally measured at different volume fractions (up to 5 v%), and also at various temperatures (up to 80°C). Stability was measured using a Turbiscan Lab Expert (Formulaction SA). Thermal conductivity was measured by the hot wire technique using a KD2 Pro (Decagon Devices, Inc.). Specific heat was measured by means of Differential Scanning Calorimetry with a DSC1 (Mettler Toledo). Viscosity was measured with a RheoStress 1 rotational rheometer (Thermo Scientific). Available models were used to fit the experimental data at the different concentrations and temperatures.

More information on preparing nanofluids, measuring the thermo-physical properties and the obtained results can be found in the previous aforementioned work [4].

**Fig 1:**
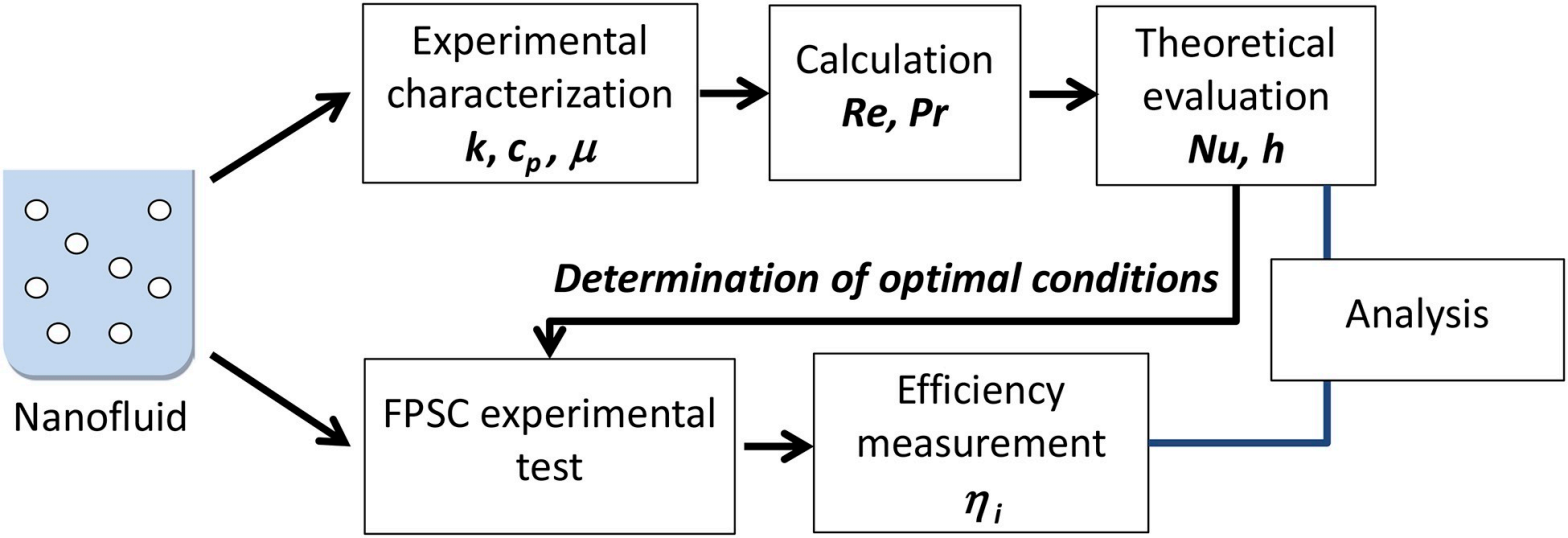
Methodology flow chart.

Fig 1 shows the methodology followed for conducting this research study.

## Experimental set-up

**Fig 2:**
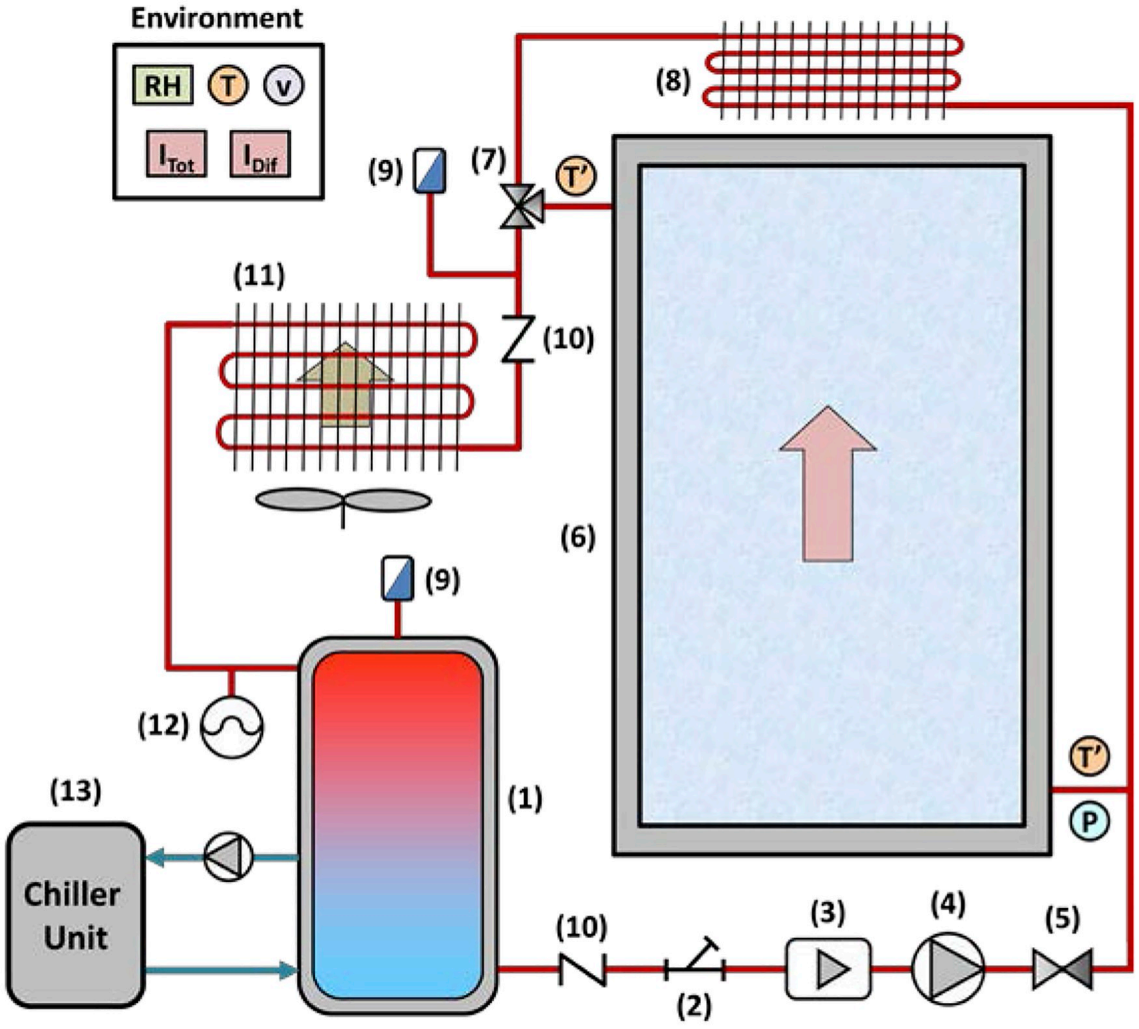
Diagram of the experimental set-up: (1) Accumulator tank; (2) filter; (3) magnetic flowmeter; (4) recirculating pump; (5) metering valve; (6) solar collector; (7) thermostatic valve; (8) passive air cross-flow heat exchanger; (9) steam trap; (10) check valves; (11) heat exchanger; (12) expansion vessel; (13) chiller.

The experimental facility used to evaluate the influence of nanoparticles on solar collector effectiveness was composed of an FPSC that worked in a close-loop, designed for water according to Standard UNE-EN 12975–2. Fig 2 presents a schematic diagram of the system, where the fluid is pumped to the FPSC by a recirculating pump installed after the insulated accumulator tank. This accumulator maintained the fluid temperature using two electrical resistors of 750 W, each controlled by a PID and an external chiller connected to the close-loop with a brazed-plate heat exchanger. Once the fluid is heated by solar radiation, it is cooled in a forced-air heat exchanger to reduce its temperature before entering the accumulator tank. The set-up was completed with a filter, two check valves, two steam traps, a metering valve to control fluid flow, and an 8-liter expansion vessel. In order to avoid excessive temperatures inside the circuit, a thermostatic valve worked at a fixed temperature of 90°C and a passive heat exchanger was used to reduce the fluid temperature, especially when the system was not running.

Table 1 summarizes the characteristics of the main elements in Fig 2. To minimize the heat exchange with the environment, the pipe from the accumulator tank to the FPSC was insulated with 6 mm-thick foam covered by an exterior aluminum coat. It is worth mentioning that the materials used in the facility have to be compatible with the tested fluids. Thus the use of galvanized steel was limited given its incompatibility with the commercial alumina nanofluid.

**Table 1:** Characteristics of the main elements.

Number	Component	Main characteristics
**1**	Accumulator tank	Volume: 80 liters Insulation thickness: 9 mm
**2**	“Y” Filter	Pore size: 500 μm
**4**	Fluid pump	Maximum flow rate: 60l·min^-1^ Maximum head: 5 mwc Power consumption: 43–82 W
**6**	Flat-plate solar collector	Absorber dimensions: 2003 x 1003 mm Inner volume: 1.15 liters Absorption area: 2.01 m^2^ Collector tilt angle: 40° Header tube inner diameter (Cu): 16 mm Raiser tubes inner diameter (Cu): 6 mm Number of raiser tubers: 10
**8**	Passive-air heat exchanger	Heat transfer surface: 1.96 m^2^
**11**	Forced-air heat exchanger	Maximum power dissipation: 24.4 kW

The measurement elements depicted in Fig 2 are summarized in Table 2, including calibration range and accuracy. All the elements were connected to a NI SCXI-1000 data acquisition system with a 30-second register time from 8 am to 9 pm. The environmental measurements, such as ambient temperature, humidity ratio, air velocity and solar radiation, were registered aside from the FPSC to avoid shadows.

**Table 2:** Transducers installed in the facility.

Measured variable	Measurement device	Calibration range	Calibrated accuracy
**Temperature**	PT100 thermoresistance	0–100°C	± 0.1°C
**Pressure**	Pressure gauge	0–10 bar	± 0.06 bar
**Volume flow rate**	Magnetic flow meter	0 to 20 l·min^-1^	± 0.25% of reading
**Velocity**	Anemometer	0–160 km·h^-1^	± 3% of reading
**Solar radiation (total)**	Pyranometer	0–2000 W·m^-2^	± 4 W·m^-2^
**Solar radiation (diffuse)**	Pyranometer	0–2000 W·m^-2^	± 4 W·m^-2^
**Temperature and relative humidity**	Combined temperature and RH transmitter	10 to 90% -40 to 80°C	± 2% RH ± 0.15°C

Tests were carried out in the city of Castellón de la Plana, Spain (latitude of 39° 59' 28.83'' N; longitude of 0° 4' 5.86'' W) in July. The experimental conditions were established according to standard UNE-EN 12975–2, which requires a constant mass flow rate of at least 0.02 kg/s per square meter of solar area, with an average temperature that equals the ambient air temperature ± 3 K. As the average temperature in July was 31.5°C, the thermo-physical properties of the fluids were evaluated at this temperature.

## Results and discussion

### Thermo-physical properties of nanofluids

The thermal conductivity, specific heat and viscosity of the alumina nanofluid were measured within a wide range of temperature and solid content and results were modeled. The results can be found in a previous work by the authors [4]. This modeling allows nanofluid properties to be determined at the temperature that the solar collector worked at all year long, which avoids needing to measure them every season.

From the previous characterization, it was concluded that the nanofluid’s thermal conductivity at all the evaluated temperatures could be calculated using the Maxwell equation with a maximum error of 1.18%:

$$k_{nf}=\frac{k_{p}+2k_{bf}+2(k_{p}-k_{bf})\phi }{k_{p}+2k_{bf}-(k_{p}-k_{bf})\phi }k_{bf}$$ (1)

where *ϕ* is the volume fraction of nanoparticles, and *k*_*nf*_, *k*_*p*_ and *k*_*bf*_ are respectively the thermal conductivities of the nanofluid, nanoparticle and base fluid. In order to obtain the nanofluid’s thermal conductivity at the operational temperature, the nanoparticle and base fluid values were obtained at that temperature from the handbook [25].

For specific heat, it was concluded that the mixture rule could be used to predict the nanofluid properties, as previously done in the literature by other authors. However for high concentrations, a deviation between the experimental and theoretical values was achieved within a 10% error.

$$c_{P,nf}=\frac{(1-\phi )\;\rho_{bf}\;c_{P,bf}+\phi \;\rho_{p}\;c_{P,p}}{(1-\phi )\;\rho_{bf}+\phi \;\rho_{p}}$$ (2)

where *ϕ* is the volume fraction of nanoparticles, *c*_*P*,*nf*_, *c*_*P*,*p*_ and *c*_*P*,*bf*_ are respectively the specific heat values of the nanofluid, nanoparticle and base fluid, and *ρ*_*p*_ and *ρ*_*bf*_ are the densities of the nanoparticle and the base fluid, respectively. In order to obtain the nanofluid’s specific heat at the operational temperature, the nanoparticle and base fluid values were obtained at that temperature from the handbook [25].

For viscosity, it was observed that Einstein’s equation, as used by other researchers, is actually limited low-volume fractions. Under these conditions, the increase in viscosity with the solid content was linear. However for higher concentrations, the increase in viscosity did not follow this trend and a different equation had to be used. For the alumina nanofluid used herein, it was concluded that viscosity could be modeled by the equation proposed by Kitano et al. [26]:

$$\mu_{nf}=\mu_{bf}\;{\left(1-\frac{\phi }{\phi_{m}}\right)}^{-2}$$ (3)

where *ϕ* is the volume fraction of nanoparticles, *μ*_*nf*_ and *μ*_*bf*_ are respectively the viscosities of the nanofluid and the base fluid, and *ϕ*_*m*_ is the maximum packing fraction that nanoparticles could achieve.

In order to obtain the nanofluid’s viscosity at the operational temperature, the base fluid’s viscosity at that temperature was obtained from the literature, and a correlation for the maximum packing fraction was obtained from the previous characterization.

$$\phi_{m}=5.10^{-6}T^{2}-4.10^{-4}T+0.118$$ (4)

Finally, the nanofluid’s density was calculated from the mixture rule:

$$\rho_{nf}=(1-\phi )\;\rho_{bf}+\phi \;\rho_{p}$$ (5)

where *ρ*_*nf*_, *ρ*_*p*_ and *ρ*_*bf*_ are the densities of the nanofluid, nanoparticles and base fluid, respectively.

**Fig 3:**
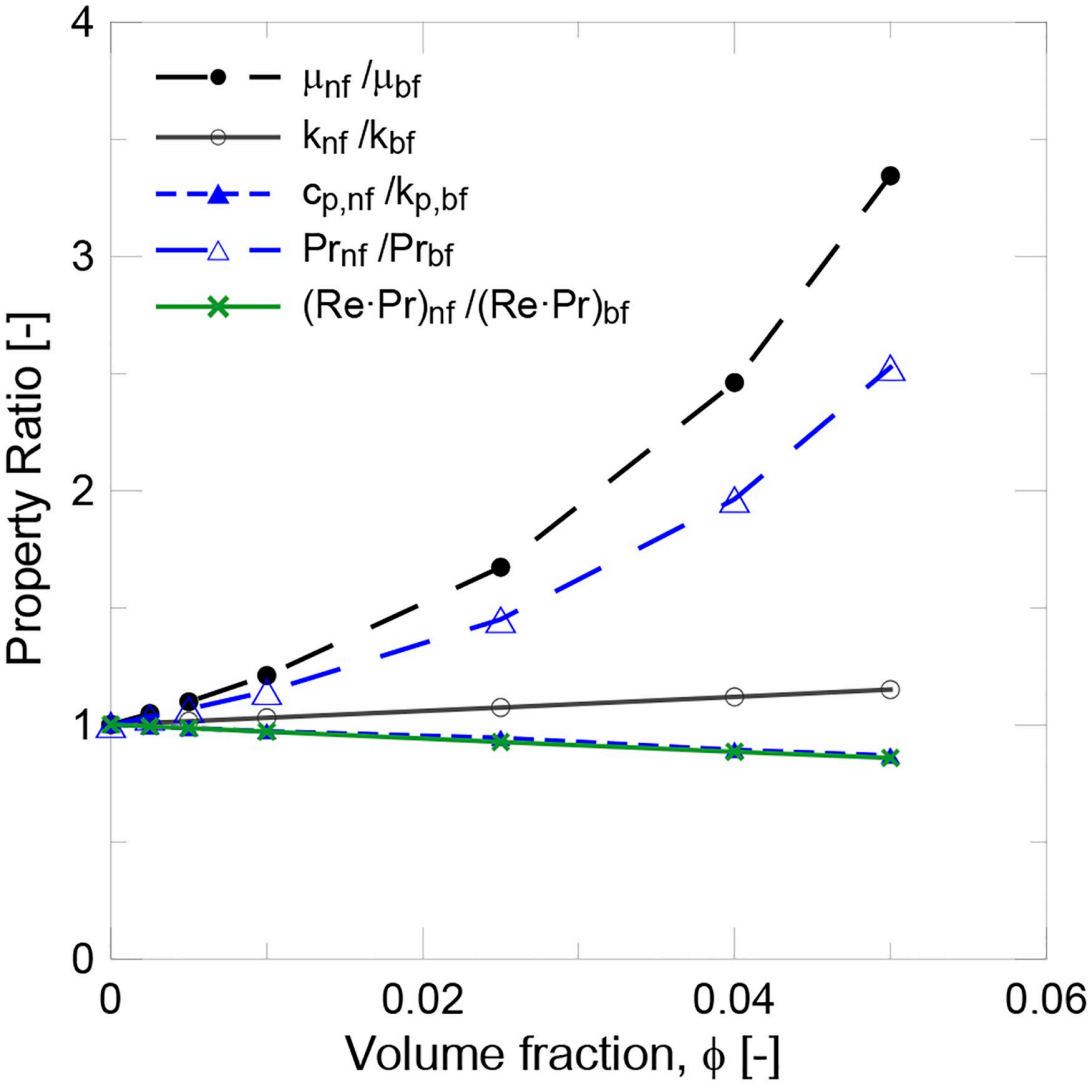
Evolution of thermal conductivity, specific heat, viscosity, and the Pr and (Re·Pr) ratios with the volume fraction.

Table 3 shows the values of the properties for water (base fluid) and the alumina nanoparticles at 31.5°C, as used to calculate the thermal conductivity, specific heat, viscosity and density of the nanofluids with different solid contents. The ratio between the nanofluid and base fluid properties is plotted in Fig 3. As expected, specific heat decreased with the solid content, while both thermal conductivity and viscosity increased. For low concentrations (below 0.5 v%),. the increase in viscosity barely exceeded the thermal conductivity enhancement. However for higher concentrations, viscosity stopped increasing linearly and the thermal conductivity enhancement was negligible compared to the increase in nanofluid viscosity.

**Table 3:** Thermo-physical properties of water and alumina at T = 31.5°C.

Sample	*k* (W·m^-1^·K^-1^)	*c*_*p*_ (J·kg^-1^·K^-1^)	*μ* (Pa·s)	*ρ* (kg·m^-3^)
**Water**	0.656	4180	7.73·10^−4^	995.21
**Al_2_O_3_**	35.4	786.17	-	3680

Fig 3 illustrates the evolution with the solid content of the Prandtl number (*Pr*) ratio, and the (*Re·Pr*) ratio is shown at the 0.24 l/min flow rate according to the standard (*v* = 0.14 m/s). The following equations were used:

$$\text{Pr}=\frac{c_{P}\cdot \mu }{k}$$ (6)

$$\text{Re}=\frac{\rho \cdot v\cdot D}{\mu }$$ (7)

$$(\text{Re}\cdot \text{Pr})=\frac{\rho \cdot v\cdot D\cdot c_{P}}{k}$$ (8)

At high concentrations, viscosity became the most important parameter to influence the Prandtl number. However, the product (*Re·Pr*) did not depend on the nanofluid’s viscosity, which was affected mainly by the drop in the specific heat capacity when adding nanoparticles.

### Heat transfer performance of nanofluids

The alumina nanofluids’ heat transfer performance was evaluated at different solid contents through the evolution of the heat transfer coefficient (*h*) in the riser tubes of the FPSC. The heat transfer coefficients were obtained from the previously calculated Nusselt (*Nu*) number values.

**Fig 4:**
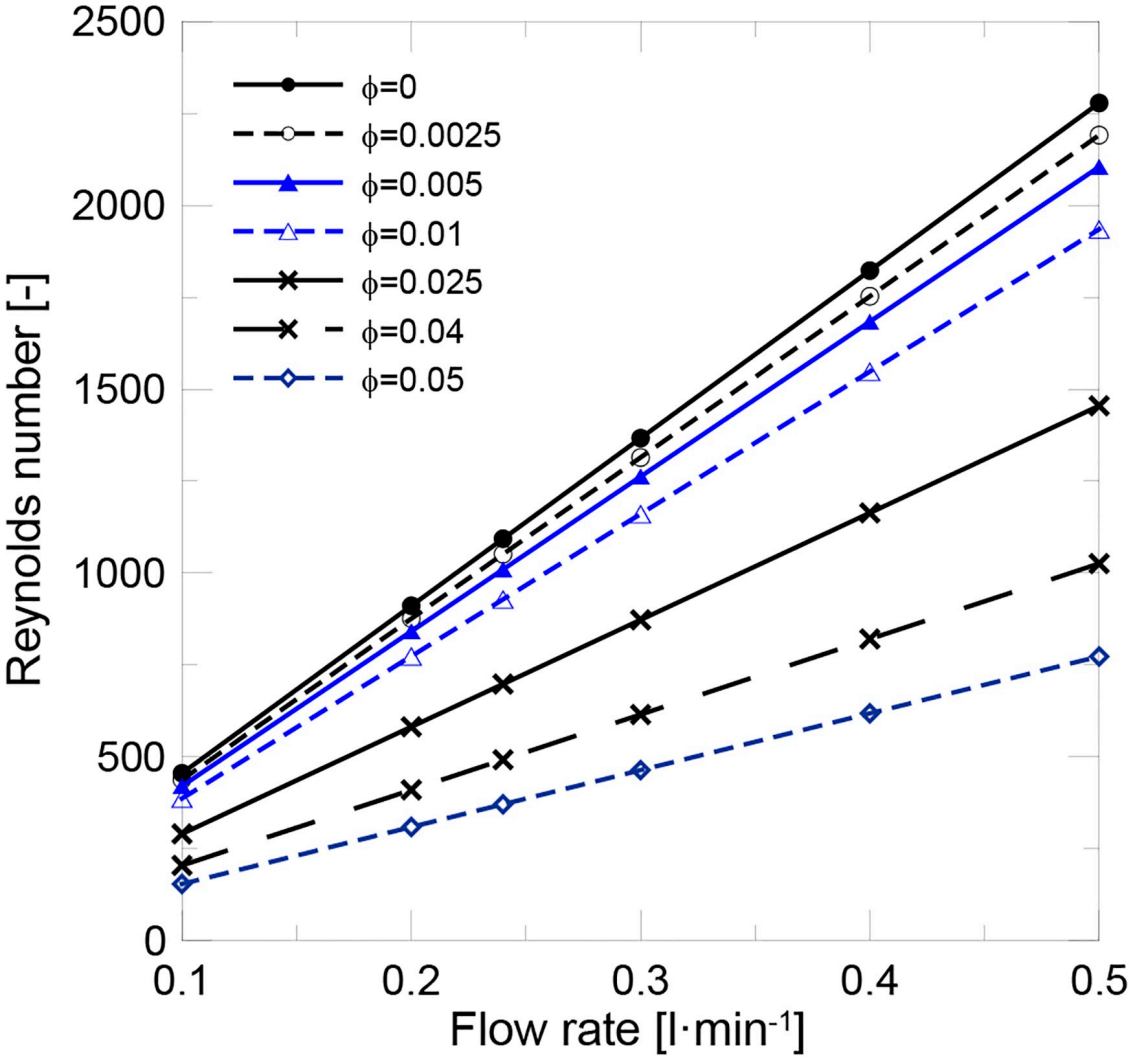
Evolution of the Reynolds number with flow rate and volume fraction.

In this work, a total flow rate of 2.4 l/min was established as the reference value for the experimental validation, with the individual flow rate for each riser tube being 0.24 l/min. Therefore, the theoretical heat transfer performance evaluation was made within a range of flow rates from 0.1 l/min to 0.5 l/min. Fig 4 shows the evolution of the Reynolds number with flow rate at different solid contents. It can be observed that at a constant flow rate, the Reynolds number lowers due to the increase in nanofluid viscosity. A laminar flow regime was achieved for all the evaluated conditions.

In the laminar flow regime, the Nusselt number is considered constant in the fully developed region. However in the entry length, the Nusselt number decays from the inlet to the fully developed conditions, where $\frac{x/D}{\text{Re}\cdot \text{Pr}}\approx 0.05$. In this work, the fully developed region was achieved at *x/L* = 0.85 for water and at *x/L* = 0.80 for the nanofluid. Hence the Nusselt number could not be assumed constant and was calculated by the following equation:

$$Nu=3.66+\frac{0.0668\cdot (D/L)\cdot \text{Re}\cdot \text{Pr}}{1+0.04\cdot {\left[(D/L)\cdot \text{Re}\cdot \text{Pr}\right]}^{2/3}}$$ (9)

The heat transfer coefficient was calculated from the Nusselt number as follows:

$$Nu=\frac{h\cdot D}{k}$$ (10)

**Fig 5:**
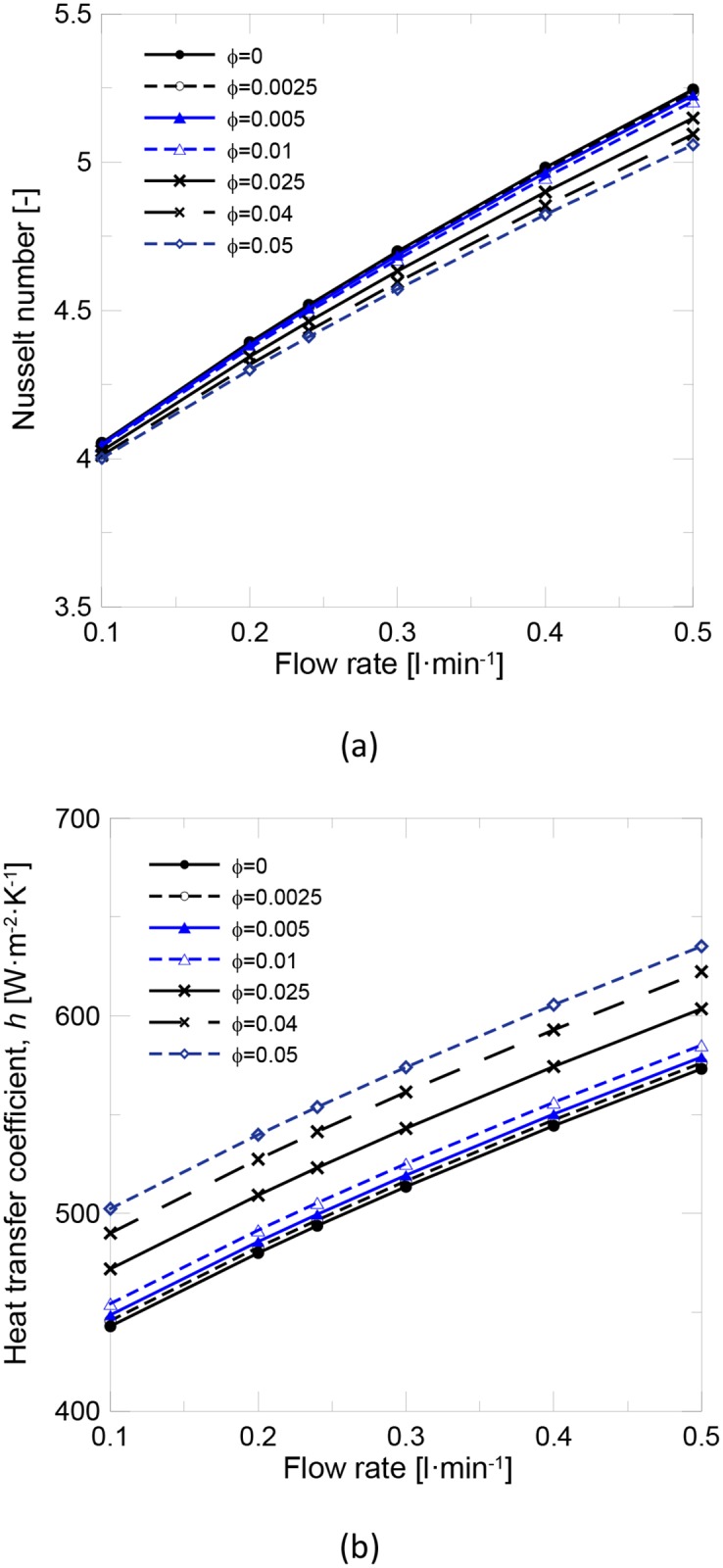
Evolution of (a) the Nusselt number and (b) the heat transfer coefficient with flow rate and volume fraction.

Fig 5(a) shows the evolution of the Nusselt number with flow rate at different solid content. It can be observed that at a constant flow rate, the Nusselt number remained almost constant and only slightly decreased for the highest concentrations. As the Nusselt number depended on the (*Re·Pr*) product, which was concluded to be affected mainly by the reduction in the specific heat capacity, no significant influence of any other thermo-physical properties was observed. Fig 5(b) shows the evolution of heat transfer coefficient. It was concluded that the heat transfer coefficient increased with solid content at a constant flow rate. As the Nusselt number remained almost constant when the solid content increased, the heat transfer coefficient became directly proportional to thermal conductivity. Consequently, the heat transfer performance in the laminar flow regime was not affected by nanofluid viscosity, and the thermal conductivity enhancement became the most important parameter to be optimized.

The ratios between the nanofluid and base fluid (*Re·Pr*), *Nu* and heat transfer coefficients at a constant flow rate of 0.24 l/min from previous figures are shown in Table 4.

**Table 4:** (Re·Pr), Nu and heat transfer ratios at 0.24 l/min.

Volume fraction, *ϕ*[–]	(Re·Pr)_nf_/(Re·Pr)_bf_	Nu_nf_/Nu_bf_	h_nf_/h_bf_
**0.0025**	0.992	0.999	1.006
**0.005**	0.984	0.997	1.012
**0.01**	0.969	0.995	1.023
**0.025**	0.925	0.988	1.059
**0.04**	0.884	0.981	1.096
**0.05**	0.857	0.976	1.122

**Fig 6:**
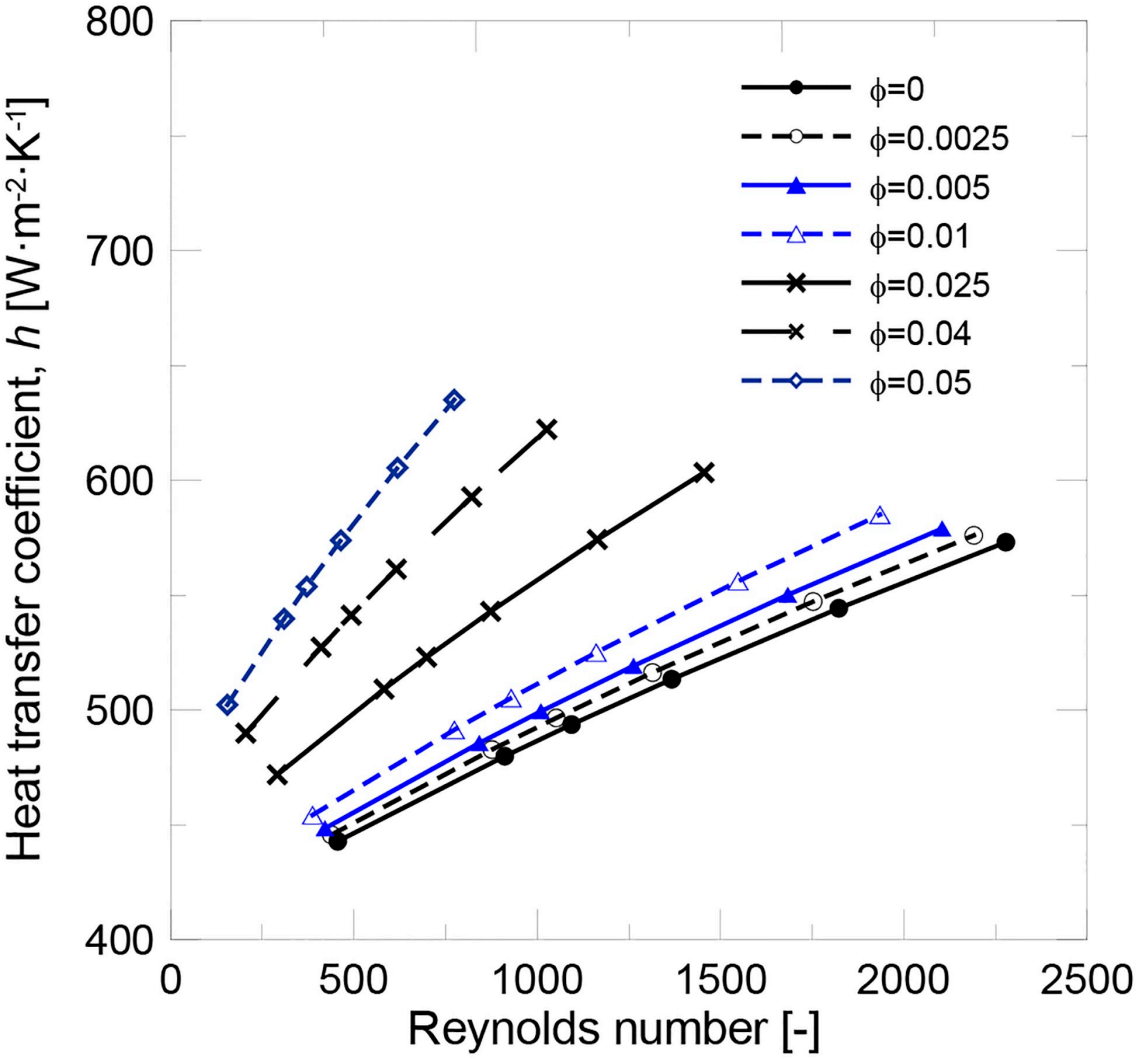
Evolution of the heat transfer coefficient with Re and volume fraction.

These results indicate that using many diluted nanofluids as the working fluid in an FPSC does not significantly increase either heat transfer or efficiency. More concentrated nanofluids have to be used to increment the heat transfer coefficient. The main drawback of using concentrated nanofluids is the increase of viscosity. Another case of study is working on a constant Reynolds basis condition. Fig 6 shows that the increment in the heat transfer coefficient is higher under these experimental conditions and a 6.36% enhancement could be achieved for the 1 v% nanofluid. The problem for keeping constant Re when the working fluid changes from water to a nanofluid is that the increase in the viscosity needs to be compensated by a rise in the flow rate and, thus, in pumping power. As in all cases, the knowledge of the thermo-physical properties is needed to evaluate its thermal performance and efficiency in applications performed under real experimental conditions.

### Thermal efficiency of the FPSC using nanofluid

The FPSC’s efficiency was experimentally measured with pure water and commercial alumina nanofluid at 1 v% of solid content. From previous calculations, the heat transfer coefficient should increase by 2.34% under the experimental condition at a constant flow rate of 2.4 l/min (0.24 l/min per riser tube).

**Fig 7:**
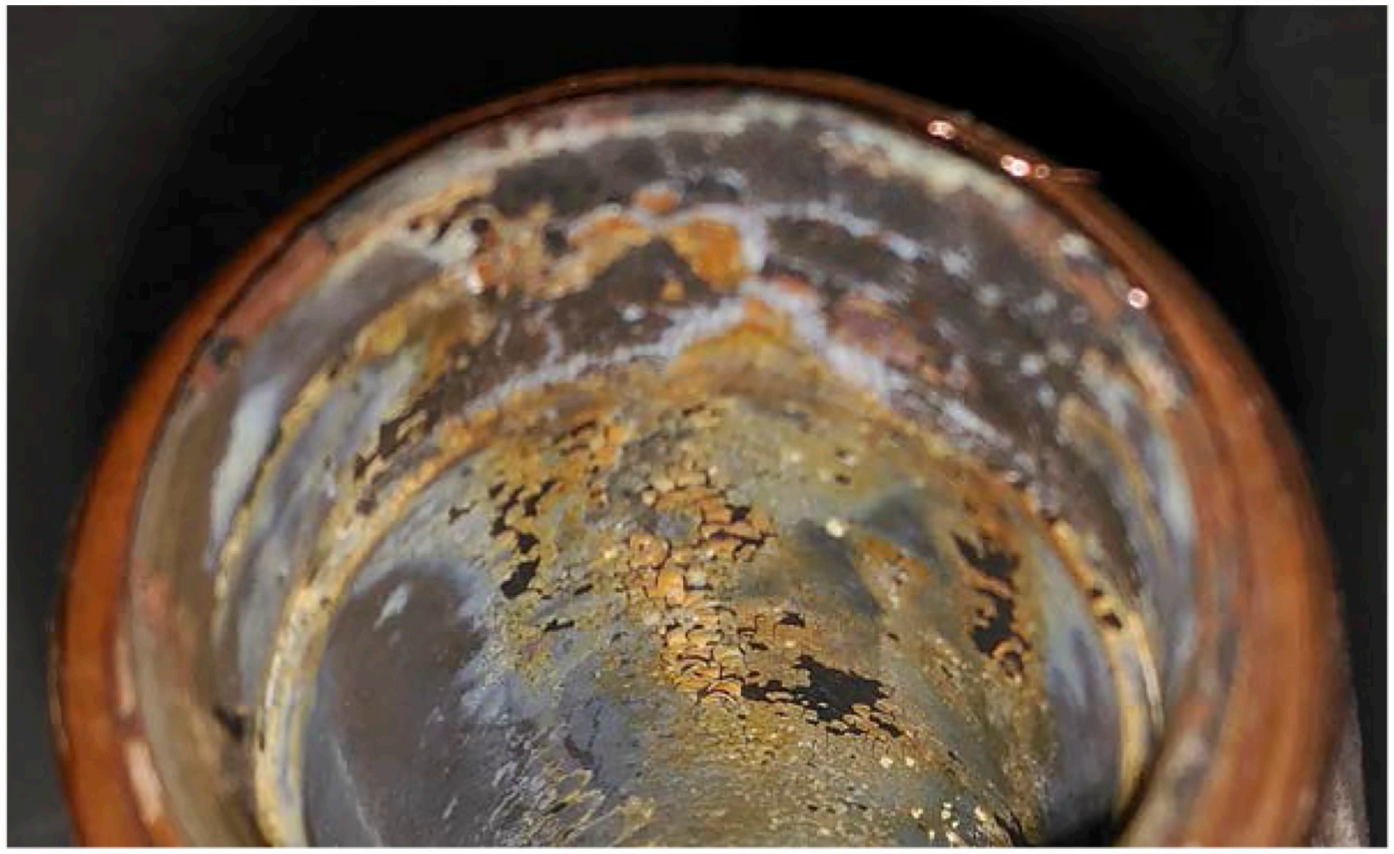
Nanoparticle deposition after the efficiency tests.

After the tests, deposition of nanoparticles was observed on the wall of the elements and pipes in the circuit (see Fig 7). This layer was formed when the nanofluid stabilized under acidic conditions came into contact with the copper hot surface, and its velocity was too low to prevent such deposition. The conditions of high temperature, small diameter and low velocity in the riser tubes are prone to increase the deposition of nanoparticles. This deposition increases with the solid content and leads to diminished heat transfer performance due to the additional thermal resistance caused by this nanoparticle layer [27]. Therefore, although the heat transfer coefficient should increase for the alumina nanofluid under ideal conditions, the solar collector’s global efficiency was expected to decrease given the formation of the deposition layer.

The instantaneous collector efficiency relates the useful energy to the total radiation incident on the collector surface by this equation:

$$\eta_{i}=\frac{Q_{u}}{A_{C}\cdot G_{T}}=\frac{\dot{m}\cdot c_{P}(T_{o}-T_{i})}{A_{C}\cdot G_{T}}$$ (11)

where *η*_*i*_ is the instantaneous collector efficiency, *Q*_*u*_ is the rate of useful energy gained, *A*_*C*_ is the solar collector’s surface area, *G*_*T*_ is the global solar radiation, $\dot{m}$ is the fluid’s mass flow rate, and *T*_*o*_ and *T*_*i*_ are the outlet and inlet fluid temperature, respectively.

The error in the experimental measurement of the instantaneous thermal efficiency was calculated by means of the propagation of error method taking into account the accuracy of the measurement sensors provided in Table 2. The values obtained for the percentage of error over the instantaneous efficiency are ranging from 9% to 15% in all the experiments with an 11.15% of mean value.

According to standard ASHRAE 93, if thermal efficiency tests are performed near the incident conditions so that *F*_*R*_(*τα*) is constant and both *F*_*R*_ and *U*_*L*_ are constant within the range of tested temperatures, a straight line will result when efficiencies are plotted against (*T*_*i*_-*T*_*a*_)/*G*_*T*_ according to the following equation:

$$\eta_{i}=F_{R}(\tau \alpha )-F_{R}\cdot U_{L}\frac{T_{i}-T_{a}}{G_{T}}$$ (12)

where *F*_*R*_ is the heat removal factor, (*τα*) is the absorptance-transmittance product, *U*_*L*_ is the solar collector’s overall loss efficiency and *T*_*a*_ is the ambient temperature.

**Fig 8:**
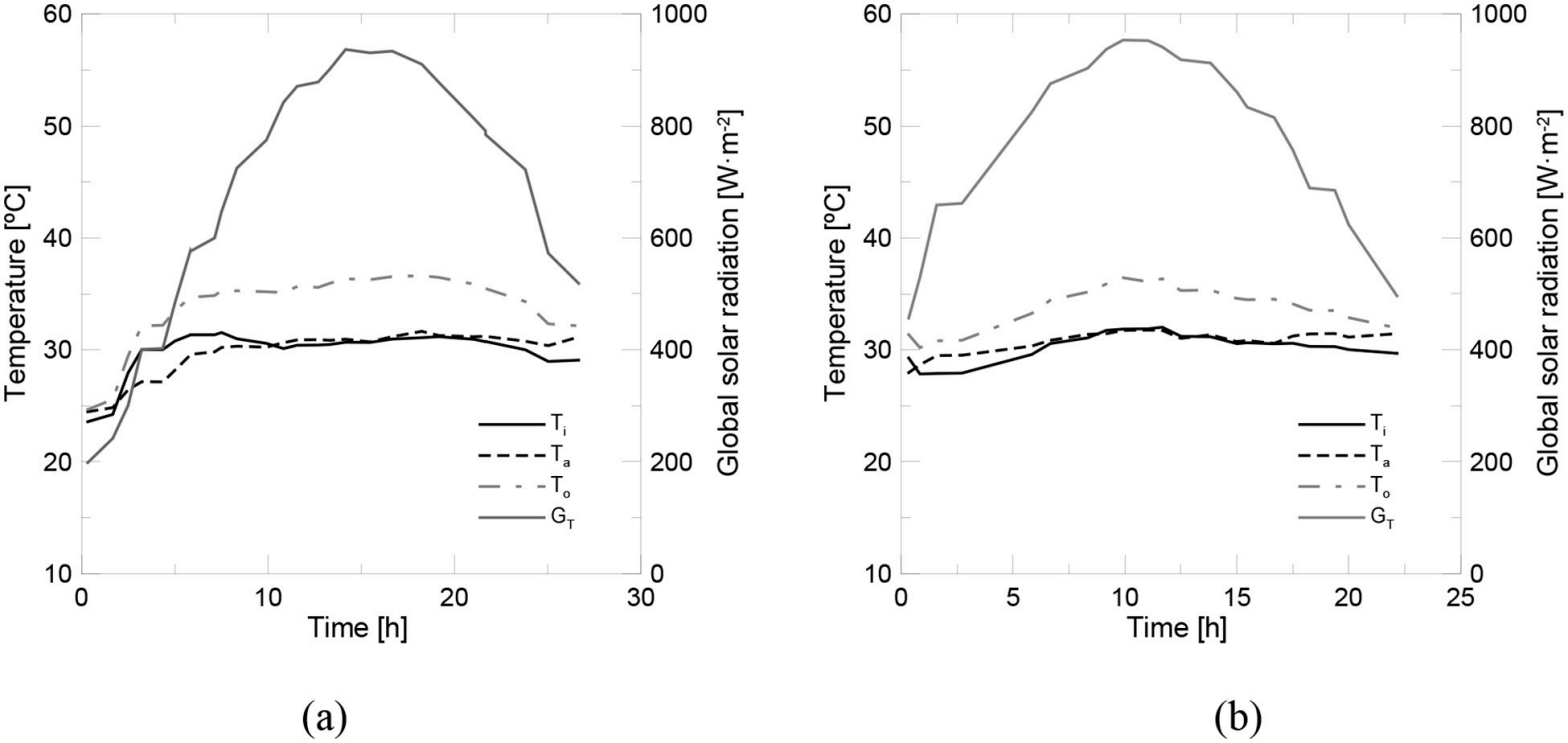
Experimental data for (a) water and (b) the alumina nanofluid at 1 v% (2.4 l/min).

**Fig 9:**
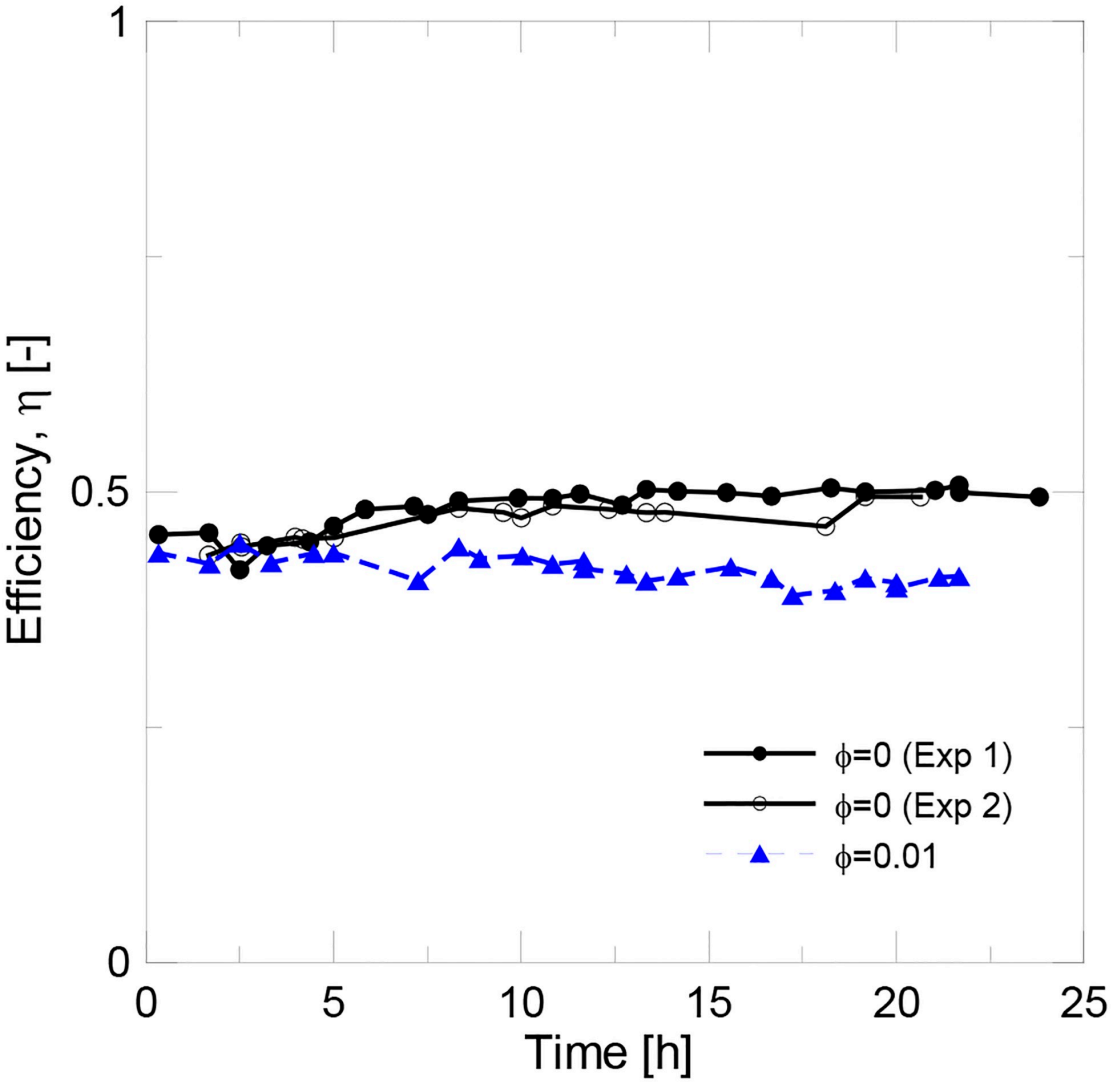
Instantaneous efficiency for water and the alumina nanofluid at 1 v% (2.4 l/min).

**Fig 10:**
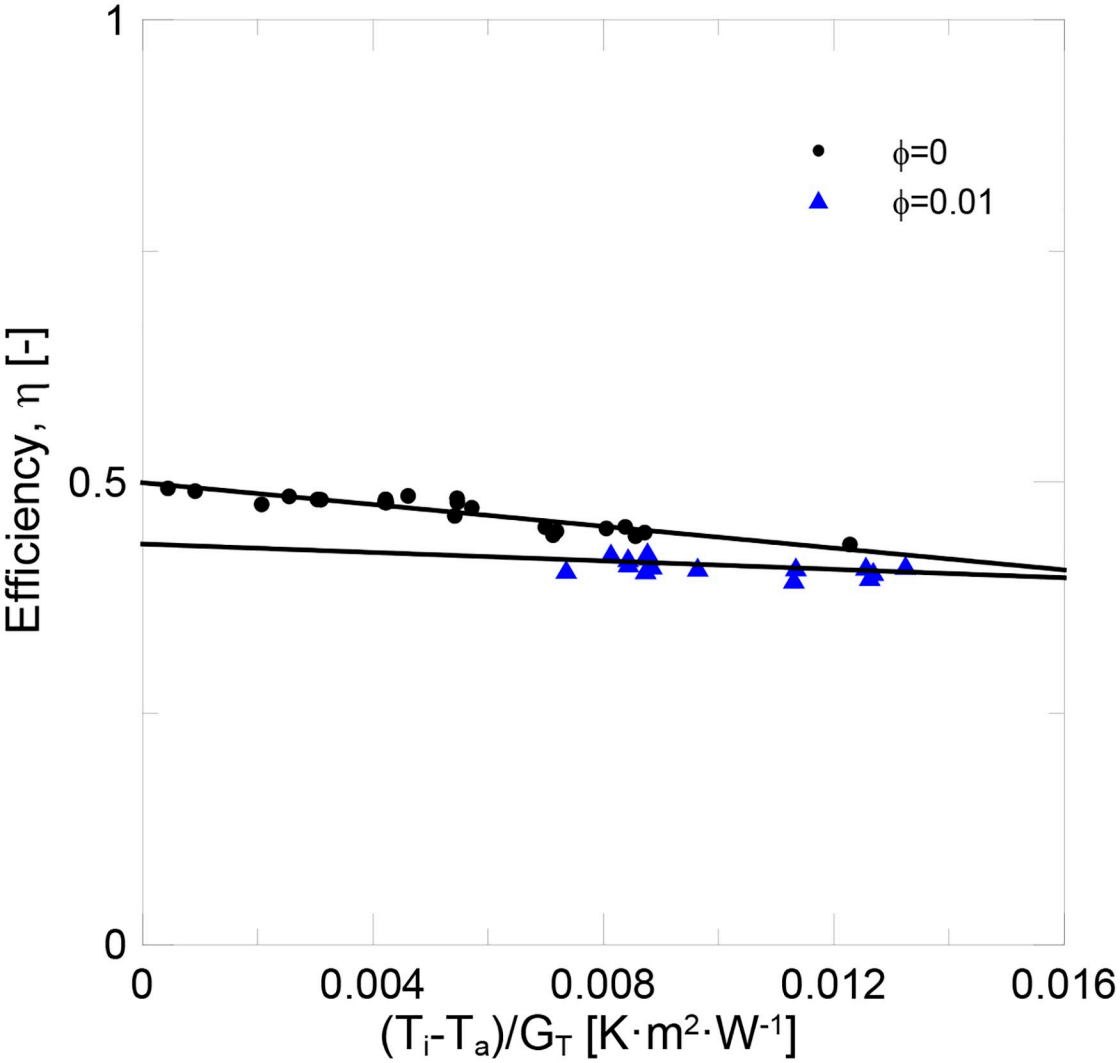
Efficiency for water and the alumina nanofluid at 1 v% (2.4 l/min).

Fig 8 shows the experimental data recorded for water and the nanofluid. The instantaneous efficiency of both samples is shown in Fig 9. Two experiments were run for pure water to check the reproducibility of the experimental tests. The solar collector’s efficiency is initially similar to water when the nanofluid was used. However, efficiency decreased with time, which suggested the formation of the nanoparticle deposition layer during the initial period of the experimental tests. The mean efficiency achieved for pure water was of 47%, while it was 41.5% for the nanofluid. In Fig 10, the experimental data were fitted to Eq 12. The results for the fitting parameter are shown in Table 5. The results obtained for pure water well agree with the previous works found in the literature [10–12, 14, 17].

**Table 5:** F_R_(τα) and F_R_·U_L_ parameters at 2.4 l/min.

Sample	*F*_*R*_*(τα)*	*F*_*R*_*·U*_*L*_
**Water (*ϕ* = 0)**	0.499	5.89
**Al_2_O_3_*ϕ* = 0.01**	0.433	2.28

## Conclusions

The purpose of this work was to evaluate the heat transfer performance of a nanofluid circulating through an FPSC from their experimentally measured thermo-physical properties, and to predict the improvement of the collector’s thermal efficiency compared to using pure water.

From the obtained results, it was concluded that the FPSC’s thermal efficiency can be theoretically improved using nanofluids as the working fluid, but only under specific experimental conditions. This work demonstrated that the Nusselt number and the heat transfer coefficient in the laminar flow regime are not affected by the nanofluid’s viscosity. Only the thermal conductivity and the specific heat capacity influence the nanofluid’s heat transfer performance.

The nanoparticle concentration needs to be increased to obtain a thermal conductivity enhancement superior to the specific heat decrement. However, concentrated nanofluids present higher viscosities and require pumping power, while the probability of nanoparticle deposition on the walls of tubes is high.

For the commercial alumina nanofluid tested at 1 v%, a constant flow rate allowed the heat transfer coefficient to be theoretically increased by 2.34% under ideal conditions, but the experimental tests done with the FPSC showed that efficiency decreased due to deposition layer formation.

In conclusion, the thermo-physical properties of the selected nanofluid need to be previously measured experimentally to evaluate its effect on the heat transfer coefficient. In the laminar flow regime, the use of nanofluids can improve thermal efficiency, but only if the nanoparticle concentration is high enough to provide a thermal conductivity enhancement, but no so high as to avoid nanoparticle deposition. Higher efficiency can be achieved by working at constant Reynolds basis but, in this case, an increase in the flow rate and pumping power are required.
